# Pathogenesis

**Published:** 1868-10-01

**Authors:** H. Lebert

**Affiliations:** Professor of Clinical Medicine of the University of Breslau


### CHICAGO MEDICAL JOURNAL.

*Vol. XXV.—OCTOBER* 1, 1868.—*No.* 19.

### PATHOGENESIS.

*The Pathological Anatomy and Pathogenesis of Disseminated Chronic Pneumonia, and of Pulmonary Tubercles. By* H. LEBERT, *Professor of Clinical Medicine of the University of Breslau.*

TRANSLATED BY WALTER HAY, M.D., ASSOCIATE EDITOR CHICAGO MEDICAL JOURNAL.

(*Continued from p.* 597.)

2nd. *Pathogeneses of chronic disseminated pneumonia and of tuberculosis.*

In casting a rapid glance over the historical development of the doctrines of tuberculosis, we find, up to the beginning of this century, an extreme confusion of ideas, and the term phthisis, which, until to-day has preserved its parasitic existence in science, was applied to the most diverse pulmonary affections.

Bayle and Laennec first associated all the alterations of the pulmonary tissue, in regarding them as originating from tubercles, and from their different phases of development. They thus introduced into science, for more than half a century, this strange confusion, prevailing even now among many pathologists, which associates in the same morbid entity pneumonic foci and true tubercles. From this epoch Broussais defended, with energy, a totally opposite doctrine. According to him these alterations are only inflammatory, and originate from the pulmonary capillaries, and principally from the lymphatics; but his descriptions were so inexact that his general views of inflammation, stamped as they were with the impress of genius, could rally around him but a small faction, although reckoning in this number some men of true talent.

Louis described the anatomical alterations and symptoms of pulmonary phthisis with such exactness that, whatever may be the changes of doctrine, his descriptions remain, and will ever remain, true. He takes, also, for point of departure, the tuberculous unity of Laennec. Cruveilhier and Bouillaud maintain, on their side, the inflammatory nature of all these affections, and Andral approximates nearly to them. The authors whom we have just cited base their doctrines upon a thorough knowledge of morbid anatomy; however, in spite of their high authority, the doctrine of tuberculous unity controls the schools. As to the structure of tubercle, some believe it amorphous, others consider it to be formed of inspissated pus. I have already stated that I have demonstrated, since 1844, whatever was erroneous in these two opinions, in reference to true tubercle, but during a long time I myself confounded with tubercle inflammatory products originating from connective issues, or from epithelium, whilst recognizing thoroughly the frequent combination of tubercle with phlegmasia. In 1850 Reinhardt revived the exclusive inflammatory doctrines, and endeavored to base it upon carefully made anatomical and microscopical studies; but two years later Virchow sought to rectify whatever was exaggerated in this method of examination, and separated true tubercle from chronic pneumonia, an opinion which he maintains still in his excellent work upon tumors, designating chronic pneumonia as scrofulous, and tubercle as lymphoid neoplasma.

Villemin, in an excellent treatise upon tubercle, supports the doctrines of Virchow by new investigations, exact and well made, and in 1865, he conceived the happy idea of inoculating and transmitting tubercle from men to animals, believing that to be able to re-establish the specific nature of tubercle, after histology had been compelled to abandon it.

Distinguished observers, such as L. Meyer, E. Wagner, O. Weber, Colberg, among the Germans; Martel, Vulpian, Hérard and Cornil, etc., among the French, adopted, with slight modification, nearly these views. Already, previously, Robin had insisted upon the connective element of tubercle, and had thus precluded the demonstration of the formation of tubercle, by proliferation of cells of the connective tissue. If the French authors have followed the impulse given in Germany, and especially by Virchow, they have, in their turn, been the first to draw a clinical advantage from these anatomical doctrines. It is thus that Hirtz, Chatain, Coursiers, Feltz, and others, have sought to establish the differential diagnosis between chronic pneumonia and pulmonary tubercles.

It is in this sense, also, that Niemeyer (of Tübingen) has asserted himself in a series of lectures, full of interest, and very well written, published, in the course of the last winter, in the *Weekly Clinical Journal*, of Berlin. On the other hand, Hérard and Cornil return rather to the old unity (theory) of Laennec, whilst describing very well the different forms of these diseases. They insist upon the intimate relations which unite chronic pneumonia and tubercles, attributing to these latter the principal part. They are right in this regard, that the characters of the differential diagnosis indicated up to this time, have not by any means all the value which has been attributed to them. Moreover, let us not prejudge, in this relation, the future of science, which is actually in process of transformation. The opinion at which I have arrived up to this time, and which I shall endeavor to justify elsewhere, and in a manner as complete as possible, is that in the chronic pulmonary maladies, reputed tuberculous, disseminated pneumonia predominates in such a manner that almost all that has been said of chronic phthisis is applicable to it, especially when one considers the changes which are effected therein when true tubercles are superadded thereto.

On the other hand, all that has been written concerning acute phthisis, applies essentially and by preference to acute and sub-acute pulmonary tuberculization, whether primitive or consecutive.

Before proceeding further, I desire to formulate into certain aphorisms, the pathogenetic results to which my investigations have thus far conducted me:

1st. Disseminated chronic pneumonia preponderates in the field, formerly so extended, of chronic pulmonary tuberculization.

2nd. I find no sufficient reasons for designating disseminated chronic pneumonia as scrofulous.

3rd. True tubercle offers, indeed, some characteristics of a product of new formation: however, its structure is so analogous to an inflammatory non-purulent cellular proliferation, that the doubtful question between its neo-plastic or inflammatory nature, inclines, in my opinion, strongly in favor of the inflammatory nature even of true tubercle.

4th. Tubercle is much more frequently the consequence, probably even the product of disseminated chronic pneumonia, than its cause. It is even probable that tuberculous granulation is frequently only an inflammatory metastasis, originating from inflection resulting from the inflammatory product of chronic pneumonia.

5th. This tuberculous granulation, probably inflammatory, in an anatomical point of view, constitutes, however, as a morbid element, a special affection different from simple inflammation.

6th. True tubercle is transmissible, from men to animals, by inoculation, or subcutaneous injection; but further investigation is necessary in order to know if tubercle alone is capable of determining multiple and inflammatory foci, which indeed have a great analogy with the tubercle of man, from which they take their origin. In expressing this doubt I base it upon experiments not yet concluded which I shall finish hereafter.

7th. True primitive tubercle is much more rare in the lungs than primitive disseminated chronic pneumonia; however, it predominates in afflictions of this sort of acute or subacute progress, and accelerates that of chronic pneumonia, when it is superadded thereto in its course.

8th. The very frequent coincidence of tuberculous, and especially old, inflammatory foci, with an acute tuberculization, speaks in favor of the hypothesis of the frequent metastatic and infectious nature of tubercle in its relation to inflammation, or to old tuberculization.

It is impossible to doubt the frequency of foci of chronic disseminated pneumonia, whose extreme frequency has been determined in the fact that it is called chronic tuberculization.

They have no specific characteristics, and if they occur in a constitution otherwise good, they may be cured, but to become subsequently points of departure of new pneumonic foci, or of acute tubercles.

Foci scattered and minute constitute frequently the form which is observed in strong and well constituted individuals. Nor is it less frequent that individuals, of previously feeble health, having become strong and well nourished subsequently, exhibit old relics of these diseases in the lungs and in the lymphatic glands. Whenever, in these cases, there may supervene a debilitating and prolonged disease, a chronic suppuration, a purulent pleurisy, a severe syphilitic infection, a prolonged dyspepsia, an obstinate diarrhœa, mental depression or protracted cases, these germs, slumbering, as it were, may awaken anew and originate serious and even fatal disease, either subacute tuberculization or chronic pneumonia.

As a general proposition, this last is most frequently connected with debility, either primitive or acquired, long and exhausting diseases. The hereditary proclivity so frequent amongst the sick, is probably based upon congenital weakness of the pulmonary tissue, and at an early period among such, one is struck by the chest, flat and narrow, especially in its superior strait, to which deformity rachitism does not appropriately predispose it. These disseminated foci once established exhibit a slow progress, the inflammatory products undergo a species of cellular death, with a dry, yellow, shriveled condition, or a destructive disintegration with an invasion of ulcerative molecular necrosis.

There results from these deposits, in addition to respiratory embarrassment and cough, a low fever, as well as progressive diminution of strength and rotundity. Inhalation of irritating mechanical particles is much more immediately injurious with this primitive morbid disposition than with a vigorous constitution; nevertheless, the pulmonary tissue having been once debilitated by prolonged catarrh, these injurious particles reach the air cells and penetrate therein, and it is then that their injurious effects are manifested more and more. Let us call to mind anew at this point, that disseminated chronic pneumonia, whether hereditary or acquired, or of mechanical origin, may attack as well the interstitial and peribronchial cellular tissue, as the air cells, which fact induces me to consider the expression chronic catarrhal pneumonia more correct than epithelial. I have already stated that I no longer admit the term scrofulous pneumonia, and I will also add that the chronic inflammations of infancy which simultaneously or successively have their location in the skin, in the subcutaneous cellular tissue, in the organs of the senses, upon the mucous membranes, in the periosteum, the bones, the articulations, with products as well plastic as suppurative or destructive, can not in my opinion be identical either with true glandular tubercles, or with the infiltration with tuberculous appearance of the superficial lymphatic glands. Nor do I any longer admit that lymph of bad quality can be the ordinary cause of these glandular alterations, since I have very frequently seen them exist without traces of anterior or concomitant irritation in the head, the body, or the organs of sense. Moreover, in the immense majority of cases of chronic disseminated pneumonia, I have observed, especially in the adult, this superficial infiltration or glandular tuberculization had not existed previously to, and did not co-exist with the pneumonia foci. Nothing, therefore, appears to authorize me to regard or even to call scrofulous these chronic foci of pneumonia. However, I should go too far to deny the possibility of their coincidence, of their etiological influence in a certain number of cases. It is not only against the exaggeration, the generalization of this fact, that I protest. I would not even desire to designate dsycrasia as the cause of these chronic pulmonary phlegmasiæ, especially if the term dyscrasia implies a bad quality of blood. This is the most changeable fluid of the economy; ever renewed, ever transformed, it is essentially transitory in its nature, and consequently one can not admit the prolonged residence, latent during years, of an hereditary morbid germ in this fluid.

We have already endeavored to demonstrate that true tubercle could indeed originate spontaneously, but that it was so often consecutive to anterior pneumonic foci, that involuntarily we came to regard it as their secondary infectious and metastatic product.

This opinion is not new. Dittrick* had already asserted that the tuberculous dyscrasia commenced with the arrival in the blood of the products of decomposition, especially inflammatory, in process of retrograde metamorphosis. Virchow adds, in referring to this mode of consideration, that in fact, after prolonged local diseases, and in the protracted phases of phlegmasiæ of slow resolution, tuberculosis may appear rapidly. Bahl † finally maintains the same mode of viewing the fact that necrosed particles of tissue may be transformed into tuberculous matter, and provoke, by absorption, miliary tuberculization.

We here arrive at an important point, but still disputed, the question of the specificity of tubercle. It is known that its structure does not imply it, and its partizans had become few, when the investigations of Villemin imparted to it a new impetus. In twenty-four experiments he has succeeded twenty-two times in rendering animals tuberculous. Amongst the first, I have repeated his experiments and have confirmed them; the same is true of Hirard and Cornil, who added two

* Virchow, Krankraft Geschwaelstez, Vol. II. p. 631.

† Zeitschroft für rationelle medicin, 1857, Newa Folge, Band. VII.

failures with chronic pneumonia. A year and a half ago, I made, with Dr. Wyss, numerous experiments upon this subject by inoculating, beside tubercle, every other species of morbid products, and by provoking, in other experiments, various irritations of the pulmonary tissue. I will not anticipate the results of these experiments which are not yet completed; but at the outset the successful inoculation of tubercle appeared to me to testify in favor of its specificity. New doubts have occurred to me since I have subsequently seen that lymphatic glands infiltrated with a thick tuberculiform matter, which Virchow and Villemin regard as of inflammatory origin, have equally produced, by inoculation, semi-transparent miliary tubercles. On the other hand, we have likewise seen granulations of tuberculous appearance originate by inoculation of all other substances than tubercle. Moreover, the structure of these products of inoculation had as much the character of a proliferation of the cells of connective tissue, as of those of epithelium, and in addition, accompanying the semi-transparent granular tubercle, we have established, in consequence of the inoculation, a yellowish hyperplastic infiltration diffused through many of the lymphatic glands, and in one case, in the the liver of a rabbit; besides tuberculous granulations, a diffuse hyperplasia of the interstitial connective tissue, such as is observed in incipient cirrhosis.

Hence there arises a double question, to my mind doutbful: that of the speciality of tubercle, and that of its inflammatory nature, which, after all my observations, appears to me probable. A fact which sustains this, is that sometimes in the midst of chronic pneumonia of interstitial and connective tissue, we meet with little grayish granulations, which are nothing else than a proliferation more dense and more circumscribed of the cells of the connective tissue in the midst of a diffuse hyperplasia. Indeed these same granulations entirely isolated, could not be distinguished, under the microscope, from true tubercle.

Observations which could be made upon serous membranes, especially upon the pleura and peritoneum, which Wagner has confirmed in case of the liver, and which Dr. Ebstein, according to an oral communication, in that of tubercular granulations of the heart, demonstrate around minute tubercles numerous very small foci, altogether microscopic of cells of connective tissue in process of multiplication. Now, these little hyperplastic foci resemble greatly those which are provoked in the cornea when its center, removed from all blood vessels, is irritated; nevertheless, one is compelled to admit this process occurring in the cornea to be inflammatory.

I have already suggested the resemblance which exists between the tuberculous granulation and that incipient not yet suppurative, of glanders, as well as with that of syphilitic gummata. It is exactly the gummy tissue which, as well as the tubercle, exhibits all the phases between the irritative process in the connective tissue, and the little tumor, apparently of new formation. I figured, some years ago, gummy tumors of the skin, of the heart, of the uterus, and of the ovaries, amongst which those of the skin exhibited the diffuse aspect and the microscopic elements of the inflammatory process, whilst the gummata of the heart and of the internal genital organs had the dull yellow aspect, and the same little shriveled cell apparent in the yellow tubercle in process of cell-necrosis, and exhibited the limitation of the tumors. When gummy tumors have been seen a certain number of times, it is easy to determine what Virchow has so well described, that there exists around some of these gummata a purely inflammatory cortical layer, vascular, composed of young connective cells, whilst towards the interior is found an infiltration of a dull yellow, tending toward disintegration, and the facility with which suppuration is established in these tumors is well known.

A fact which militates in favor of the inflammatory nature of gummata, is, that I have encountered in the muscles and in the brain, gummy infiltrations, altogether diffused, not presenting in any manner the sharply defined limits of tumors. The analogy of tubercle with inflammatory products finds again support in the essentially transitory character, and in the tendency to disintegration, to cellular death of true tubercle. Since even when large collections of granulations form considerable masses, as in the brain, for example, there is perceptible alongside of the irritative and hyperplastic process on the surface of the disintegrating mass, softening, fatty alteration, in proportion as they advance from the center or approach older tubercles.

Does the probably inflammatory nature of tubercle exclude it from the possession of special, not to say specific characteristics? I think not; for many infectious or contagious maladies, such as blennorrhagia, urethritis, syphilis, glanders, the small pox virus, are evidently inflammatory as regards the local disease, and yet contain an inoculable principle. It should be distinctly understood that from debilitating etiological elements a certain weakness of structure gives origin to a local irritative hyperplastic process, and consequently, those who fear new doctrines who might be designated *neophobes*, may be re-assured about the treatment of these diseases, when the limits of the inflammations extend themselves, and when the inflammatory character of all that which is to-day designated acute or chronic tubercle is established. Let those equally who behind these doctrines see the spectre of vampirism, re-assure themselves also. An irritable condition which so frequently originates from debility of the tissues or of the entire body, could only under exceptional circumstances justify antiphlogistic treatment. But on the one hand, the clearest appreciation of these morbid products, and of their locally irritable nature would have this good result, to put a curb upon the abuse of exciting methods of treatment, and of a too abundant and too analeptic regimen in diseases in which the perfect condition of the general nutrition should preoccupy the practitioner, but without leading him into exaggeration. I would add, finally, that the more carefully the nature of these pulmonary diseases shall be studied, the earlier will they be recognized, and more truly rational and salutary will their medical treatment become.

